# Universality in ant behaviour

**DOI:** 10.1098/rsif.2014.0985

**Published:** 2015-01-06

**Authors:** Kim Christensen, Dario Papavassiliou, Alexandre de Figueiredo, Nigel R. Franks, Ana B. Sendova-Franks

**Affiliations:** 1 Department of Physics, Imperial College London, Prince Consort Road, London SW7 2AZ, UK; 2 Centre for Complexity Science, University of Warwick, Coventry CV4 7AL, UK; 3 Department of Mathematics, Imperial College London, Queen's Gate 180, London SW7 2AZ, UK; 4 School of Biological Sciences, University of Bristol, 24 Tyndall Avenue, Bristol BS8 1TQ, UK; 5 Department of Engineering Design and Mathematics, University of the West of England, Frenchay Campus, Coldharbour Lane, Bristol BS16 1QY, UK

**Keywords:** social systems, behaviour, universality, controlled experiment, ant

## Abstract

Prediction for social systems is a major challenge. Universality at the social level has inspired a unified theory for urban living but individual variation makes predicting relationships within societies difficult. Here, we show that in ant societies individual average speed is higher when event duration is longer. Expressed as a single scaling function, this relationship is universal because for any event duration an ant, on average, moves at the corresponding average speed except for a short acceleration and deceleration at the beginning and end. This establishes cause and effect within a social system and may inform engineering and control of artificial ones.

## 1. Introduction

Social systems pose a major challenge in terms of planning and prediction [1,2]. Universality in relationships at the social level, such as between the size of cities and measures of income, innovation and even the pace of life [3,4] are stimulating the development of a unified theory of urban living [5]. It is not clear, however, whether universal relationships exist within societies because behaviour varies both among and within individuals [6] and involves scale invariant spontaneous activity [7–13]. Here, we show the existence of a universal relationship between the duration of an activity event and the average event speed for individuals within complete ant societies, which are model social systems [2,14]. Our results demonstrate that the average event speed profile within a society could be recovered for any event duration and corresponding environmental conditions, using a single scaling function and the value of the exponent characterizing the environmental size. This elucidates causal relationships in the workings of biological social systems and may inform the engineering and control of artificial ones.

Ant colonies are widely recognized as an experimental model for dynamical nonlinear systems [2,14], because they are self-organized adaptive societies whose macroscopic (colony-level) properties originate from interactions at the microscopic level among the individual ants themselves and between individuals and the environment. Furthermore, ants are, by any measure, extremely successful. For example, it is estimated that the biomass of ants equals the biomass of humans [15].

Why are ants so successful? Ants, like humans, are highly social. However, most human organizations have a top-down structure, that is, rules are passed down from above with the intention to control the dynamics at different levels and obtain specific outcomes. This often gives rise to rigid organizations that cannot easily adapt or self-regulate. A top-down structure might be successful in a static environment but it may be fatal in a dynamic environment. By contrast, ant colonies have a bottom-up structure. That is, ants react to local information rather than having rules imposed from above [16]. This gives rise to highly adaptive societies that can easily self-regulate [16]. Indeed, one might hypothesize that the self-organizing bottom-up structure is the generator of the proven viability of ant colonies in ever-changing environments over 100 million years [15]. Hence learning how ant colonies work is imperative.

The first prerequisite is a well-defined characteristic to describe colony dynamics. The activity of individual ants is intermittent. They stop and go. Hence, their behaviour can be described in terms of bouts of activity juxtaposed with periods of inactivity. Here, we define an activity event as a consecutive sequence of movements with non-zero speeds bounded by zero speeds. We quantify such bouts of activity in terms of their duration and associated average speed. This approach of revealing the operational principles of dynamical systems based on defining an event and quantifying it, is analogous to that applied to the study of, for example, the atmosphere and the brain. In the former, an event, such as rainfall, is quantified by its duration and associated total precipitation [17,18]. In the latter, the event is a cluster of fMRI-measured brain activity in time and space, quantified by its total size [19].

Here, we measure the activity events generated by randomly sampled individual ants tracked over 100 min within their complete colonies housed in each of two nest sizes. We find that the duration of activity spans almost 3 orders of magnitude, from seconds to minutes.

Astonishingly, the average speed of an event increases with its duration. Mathematically, the average speed of an event is a sub-linear power law of its duration. The exponent of this relationship is greater when the colony resides in a larger nest. This means that for a particular speed, the corresponding event duration is longer in the smaller nests. It suggests that the event duration is the ‘cause’ and event speed the ‘effect’.

Intriguingly, despite the variability related to nest size, there is an overarching commonality among events of different duration across nest sizes and colonies. This commonality is revealed when speed and time within an event are expressed in appropriate unitless forms. When the speeds within an event are expressed in units of the average speed for this event and time as a proportion of the event duration, the relationship between such renormalized event speeds and renormalized time coalesces onto a universal function. According to this universal function, the renormalized speed is constant and at its highest in most of the range for renormalized time except for a small acceleration and deceleration at the beginning and end. Thus, the universal function demonstrates that ants, on average, reach the characteristic average speed for an event duration almost immediately and maintain it throughout until a short deceleration at the end.

## 2. Methodology

We investigate the dynamics of the ant *Temnothorax albipennis*, which forms small colonies in rock crevices. Its natural homes are closely approximated in the laboratory by a 1 mm-high nest made of a rectangular chamber with a 2 mm-wide entrance cut out in cardboard and sandwiched between two microscope slides. The glass roof allows direct observation of the colony inside. The nest resides in a 100 × 100 mm^2^ Petri dish where food and water are available at will. Each of three colonies, C^1^, C^2^ and C^3^, was video recorded within two nest sizes: 35 × 28 mm^2^ and 55 × 44 mm^2^ in a randomized order. All six experiments were carried out within 5 days (17–21 July 2006; table 1). The colonies were collected on 9 June 2006 from Dorset, UK, and workers were individually marked with unique combinations of colour paint dots.

**Table 1.** The number of worker ants, brood and randomly sampled worker ants in colonies C^1^, C^2^ and C^3^ in the two nest sizes 35 × 28 mm^2^ and 55 × 44 mm^2^; the * denotes the first nest-size treatment. All colonies had a single queen. Any decrease in brood no. within the 5 days of the study was due to either the disappearance of eggs, which are eaten sometimes, or to pupae turning into young adults, which are immobile initially.

colony	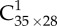	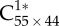	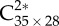	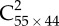	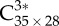	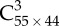
total no. ants	121		92		67	
nest size (mm^2^)	35 × 28	55 × 44	35 × 28	55 × 44	35 × 28	55 × 44
total no. brood	59	77	44	40	42	28
tracked ants	28	13	22	17	9	12

The dynamics were recorded for 100 min by a digital video camera mounted above the nest. On the video recordings, ants were tracked manually with a cursor using AntTracker v. 0.1 software [20]. This produced tracks (*x_i_*, *y_i_*; *t_i_*), where *x_i_*, *y_i_* is the position of the ant's petiole (middle) as a percentage of the video screen at time *t_i_* with time intervals of Δ*t*^(1)^ = 0.100 ± 0.001 s.

We tracked the movement of individual ants within their complete, intact colony inside laboratory nests that closely approximate their nests in the field. None of the interactions that naturally occur in these colonies were filtered out or excluded in any way. Therefore, the recorded and analysed behaviour of individual ants is subject to interactions.

Individual ants were tracked one at a time by playing back the video recording in real time. Ants were selected for tracking at random from different regions of the nest at the beginning of the recorded period. Only a small proportion of the tracked ants (on average ≈1/4) moved at the same time. Indeed, in general ants within their colony nest spend most of their time not moving. Ants that left the nest during the recorded period could not be identified reliably on their return. For this reason, the tracking of an ant was terminated on its leaving the nest.

## 3. Data processing

The data were processed to convert the percentage coordinates to spatial coordinates in units of millimetres. Manual tracking with a cursor is not ideal and small deviations from the true path of an ant are inevitable, particularly when the ant is moving fast. Furthermore, tracking on what is a pixelated computer display introduces quantization effects, where the very short time interval Δ*t*^(1)^ = 0.100 s between readings often means that only a small neighbourhood around a given point is visited. Thus, changes in the spatial coordinates are integer multiples of some minimum length scale defined by the size of a pixel. Both of these errors in the data result in fluctuations on a small length scale. To minimize their effect, we averaged out such small-scale fluctuations by applying the technique of coarse-graining well known from statistical physics [21]. The original data (*x_i_*, *y_i_*; *t_i_*) were coarse-grained by a factor *n* to produce new data points. The first new data point is3.1a
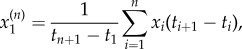
3.1b
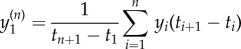
3.1c
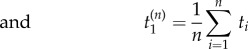
and similar for the *k*th data point 
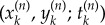
. The new time interval between data points is Δ*t*^(*n*)^ = *n*Δ*t*^(1)^.

We say that an ant is moving in the unit time interval 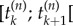 when the associated speed3.2
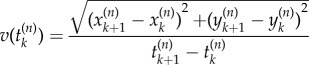
is non-zero, where 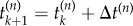. Since a period of activity (movement) is followed by a period of inactivity (stoppage) and vice versa, we can define an activity event as a sequence of *m* consecutive non-zero speeds. To be explicit, the sequence … , 0, *v*(*t*_0_), … ,*v*(*t*_0_ + (*m* − 1)Δ*t*^(*n*)^),0, … with *v*(*t*_0_ + *j*Δ*t*^(*n*)^) ≠ 0 for *j* = 0, … , *m* − 1 constitutes an activity event with duration *T* = *m*Δ*t*^(*n*)^ starting at time *t*_0_ and ending at time *t*_0_ + *T*.

In the present analysis, we coarse-grained the data to a unit time interval Δ*t*^(8)^ = 0.8 s. We chose 0.8 s as our time unit because it gives a reasonable compromise between minimizing the quantization effects due to the pixel nature of the images and minimizing the loss of information. To investigate directly the effect of the level of coarse-graining, we applied the same analysis to the data after coarse-graining to a unit time interval Δ*t*^(*n*)^ = *n*Δ*t*^(1)^ for *n* = 2, 4, 16 and 32, that is, Δ*t*^(*n*)^ = 0.2, 0.4, 1.6 and 3.2 s, respectively. We found that our results are robust to such a change in the time unit. See the end of §4 for details.

The bins associated with event durations *T* were determined according to the following reasoning. Because the original tracks had Δ*t*^(1)^ = 0.100 ± 0.001 s and the data were coarse-grained to a time unit of 0.8 s, the event durations were highly concentrated around multiples of Δ*t*^(8)^ = 0.8 s. Hence, we quantized the event duration *T* in units of 0.8 s. For example, events with duration in the interval [9.2 s,10.0 s) were assigned *T* = 9.6 s, while events with duration in the interval [10.0 s,10.8 s) were assigned *T* = 10.4 s and so on. The same reasoning applies for coarse-graining to 0.2, 0.4, 1.6 and 3.2 s with concentrations and quantization at the respective time interval.

## 4. Results: experiments

**Figure 1.**
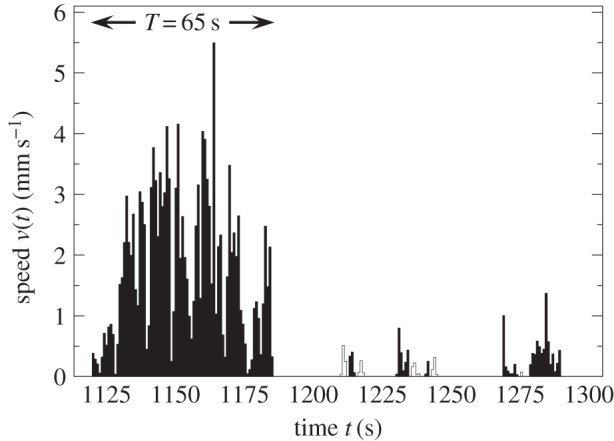
An extract of 180 s of the time-series of the speed of a 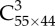 ant. An event is a consecutive sequence of non-zero speeds. There are 11 events and every other event has been coloured black. The longest event has duration *T* = 65 s. The longer the event, the higher the speed obtained.

**Figure 2.**
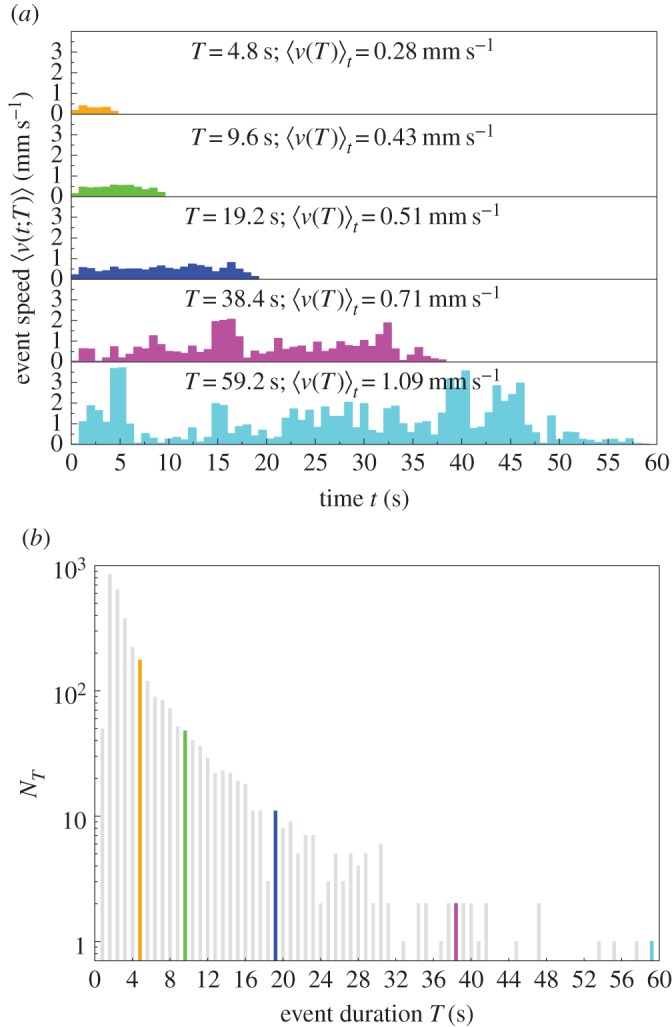
(*a*) The event speed profile: speed 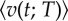 versus time *t* averaged over events with duration *T* for sampled ants in 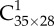, see equation (4.1). The event speed profiles have durations *T* = 4.8 s (orange), 9.6 s (green), 19.2 s (blue), 38.4 s (magenta) and 59.2 s (cyan), and the number of events *N_T_* is 176, 48, 11, 2, 1, respectively. The longer the event, the higher the average event speed 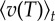, see equation (4.2). (*b*) The number of events *N_T_* versus event duration *T*. The number of events decreases with event duration that is quantized in units of 0.8 s. The highlighted events are those displayed in (*a*). The total number of events ∑*_T_N_T_*= 3219 for 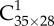.

When an ant has a longer activity event, its speed is higher (figure 1). We consider all *N_T_* events of duration *T* for the sampled ants within each colony and nest size. We evaluate the speed at time *t* for events with duration *T* by averaging over all *N_T_* events:4.1
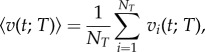
where *v_i_*(*t*; *T*) is the speed of event *i* with duration *T* at time *t*. We denote the graph of 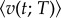 versus time *t* as the event speed profile. The event speed at time *t*, 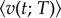, is non-zero for 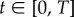. Despite the fluctuations due to the relatively small sample sizes, qualitatively, the event speed profiles show that the longer the event, the higher the speed 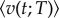 (figure 2).

To quantify how speed increases with event duration in a given colony and nest size, we consider the average speed for events with duration *T* given by4.2
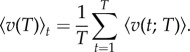


**Figure 3.**
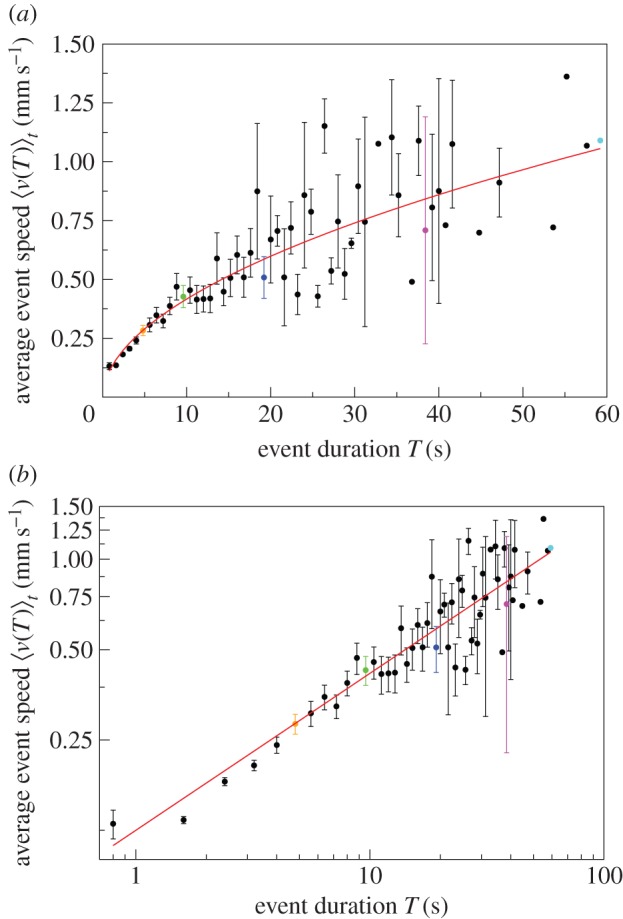
Average event speed 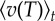 versus event duration *T* on (*a*) linear–linear scale and (*b*) log–log scale. Data for 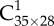 (solid circles). Error bars indicate 1 s.e. of the mean. The longer the event duration, the bigger the error bars owing to fewer events. Data points without an error bar consist of just one event. The highlighted events are the same as in figure 2. The red curve displays a power-law relationship 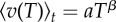, with *β* = 0.52 and *a* = 0.13, that is, the average event speed increases sub-linearly with the duration of the event.

The relationship between average speed and event duration is consistent with a power-law increase4.3
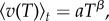
with *β* = 0.52 (figure 3). That 0 < *β* < 1 implies that the average event speed increases sub-linearly with the duration of the event.

In all six experiments, the exponent *β* is greater than zero (table 2, the 95% CIs do not overlap 0). Over all colonies, the exponent is statistically significantly higher for the larger nest size (*β* = 0.60) than for the smaller nest size (*β* = 0.47, *t* = 3.911, *n* = 340, *p* < 0.001, general linear mixed model, electronic supplementary material, tables S1 and S2, figures S1–S5) but there is no significant difference in the constant *a* (0.12 and 0.13, for the large and small nest size, respectively, *t* = −0.034, *n* = 340, *p* > 0.05, general linear mixed model, electronic supplementary material, tables S1 and S2, figures S1–S5). This suggests that the environment feeds back into the relationship between average event speed and event duration.

**Table 2.** The estimate and 95% confidence intervals of the exponents *β* and coefficients *a* (in units of mm s^−1^) associated with the power-law relationship 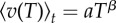 for the three investigated colonies in each of the two nest sizes. The values are based on simple linear regression fitted to the log–log relationship between average event speed and event duration.

colony	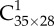	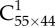	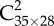	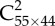	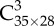	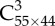
exponent *β*	0.52	0.61	0.40	0.53	0.48	0.68
95% CI for *β*	±0.06	±0.09	±0.07	±0.06	±0.12	±0.11
coefficient *a*	0.13	0.10	0.13	0.15	0.12	0.13
95% CI for *a*	±0.03	±0.03	±0.03	±0.04	±0.04	±0.04

**Figure 4.**
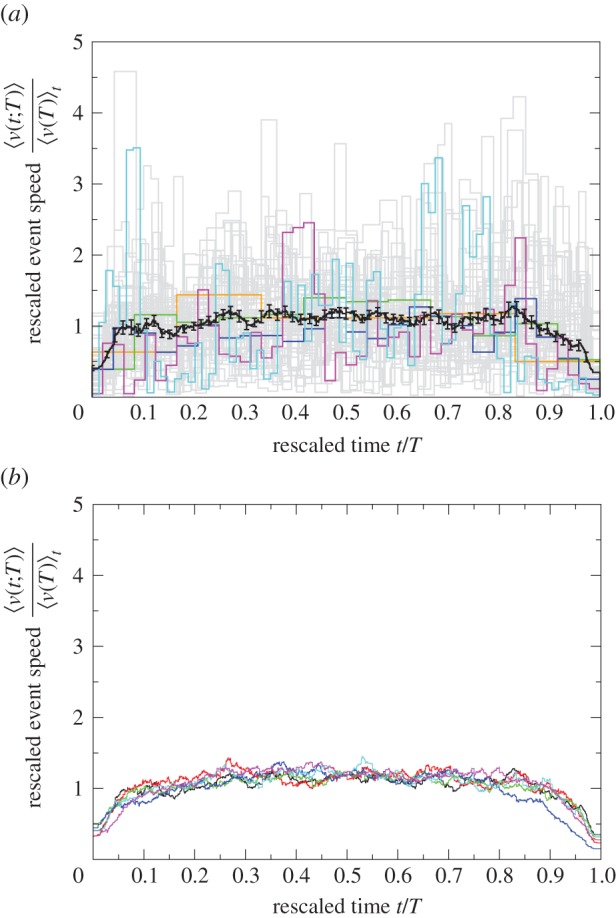
Rescaled event speed 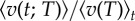 versus rescaled time *t*/*T*. (*a*) Data for colony 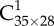. Rescaling the speeds in an event of duration *T* with its average speed aligns the event speed profiles. Grey lines represent the rescaled event speed profiles for the different durations. The highlighted events indicated with orange, green, blue, magenta and cyan are the same as in figure 2. The average rescaled event speed profile (black curve) traces out the scaling function 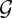 in equation (4.4). Error bars indicate 1 s.e. of the mean. (*b*) The scaling functions 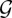 for experiments 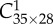 (black), 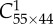 (red), 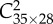 (green), 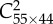 (blue), 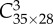 (cyan) and 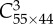 (magenta). 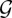 is universal because it is independent of colony and nest size.

Despite such environmentally related variability in the exponent that characterizes the relationship between the event duration and the average event speed, there is an underlying universality in event speed profiles. We express the event speed profiles of duration *T* in units of its associated average speed 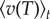 and time *t* in units of the duration such that 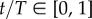 for all event speed profiles. Mathematically, this is4.4
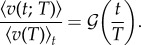
When 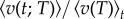 is plotted against *t*/*T*, the data coalesce and trace out the graph of the scaling function 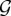. This function initially increases and then, around *t*/*T* ≈ 0.05, crosses over to a constant ≈1 before decreasing at *t*/*T* ≈ 0.90 towards zero (figure 4).

**Figure 5.**
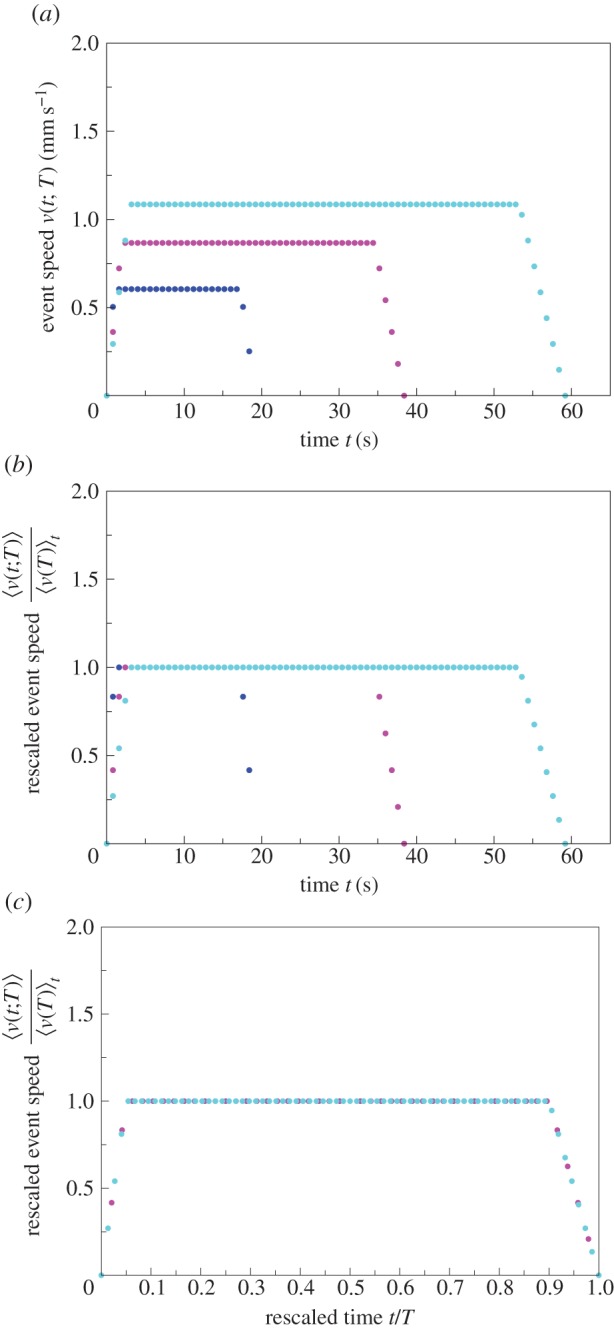
Sketch of the procedure for data collapse using a diagrammatic representation of event speed profiles obtained for colony 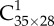.

This function reveals the commonality between event speed profiles and contains the information necessary to generate the event speed profile for any duration *T* using equation (4.4) (figure 5). In figure 5*a*, there are three different events of duration *T* = 15, 30, 60 s, respectively, where the reached constant speed increases with the event duration like *aT^*β*^* with *a* = 0.13 and *β* = 0.52 (table 2). Figure 5*b* shows that rescaling speed by dividing it by 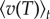 aligns vertically the three graphs. Figure 5*c* demonstrates that rescaling time by dividing it by *T* aligns the three graphs horizontally and we obtain a data collapse onto the graph of the scaling function 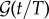. The scaling function increases until at *t*/*T* = 0.05 it reaches the constant value of 1 before starting to decrease towards zero at *t*/*T* = 0.90. Note that this process can be reversed, that is, from the graph in figure 5*c*, we can multiply the argument (*t*/*T*) of the scaling function 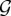 by *T* = 15, 30, 60 s, respectively, to obtain figure 5*b* and then multiply the function value 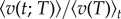 by 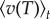 to recover figure 5*a*. Hence, the scaling function 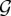 compactly contains all the information of the three different event speed profiles displayed in figure 5*a*.

The above results obtained from data coarse-grained to a time unit of Δ*t*^(8)^ = 0.8 s were replicated when the data were coarse-grained to time units of 0.2, 0.4, 1.6 and 3.2 s (electronic supplementary material, tables S3 and S4). The power-law relationship between mean event speed and event duration as well as the significant difference between the exponents for large and small nest were robust to these changes (electronic supplementary material, tables S3 and S4).

## 5. Null model

**Figure 6.**
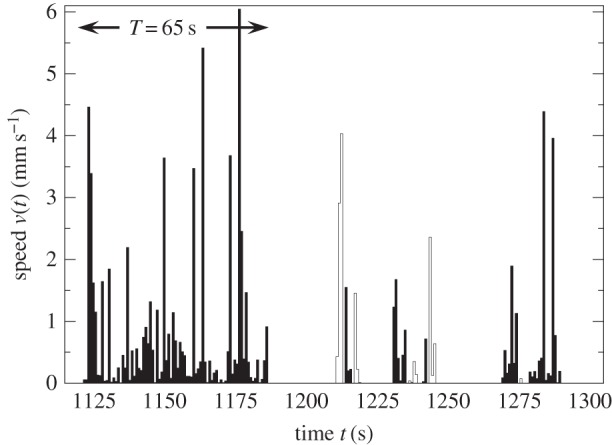
Data randomized according to the null model where all speeds are pooled together and redistributed randomly among all events without replacement. The relationship between speed and event duration is lost both within and between events. Within events, there is no structure of speeds such that they are low at the beginning and the end of the event. Between events, there is no positive relationship between speed and event duration.

As a complementary way of demonstrating that there is a non-trivial relationship between event duration and speed, we define a null model in which the observed speeds from the ∑*_T_N_T_* events are reallocated at random without replacement to each event according to its duration (figure 6). Then we recalculate everything as with the real data.

## 6. Results: null model

**Figure 7.**
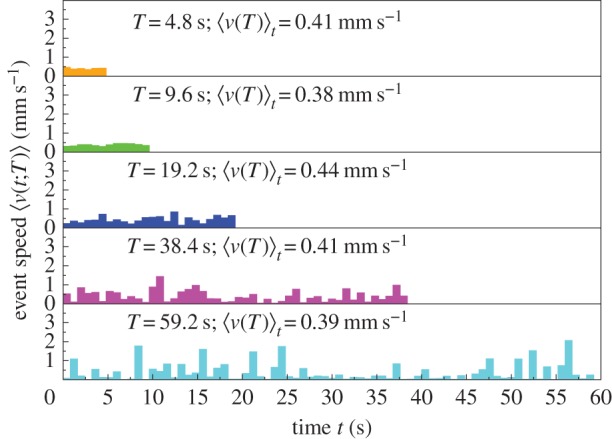
Data randomized according to the null model. The event speed profile, that is, speed 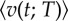 versus time *t*, has been averaged over all events with a given duration *T* in colony 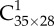. The positive relationship between average speed and event duration is lost. The average speed is constant at 0.41 mm s^−1^ for all event durations (figure 8).

**Figure 8.**
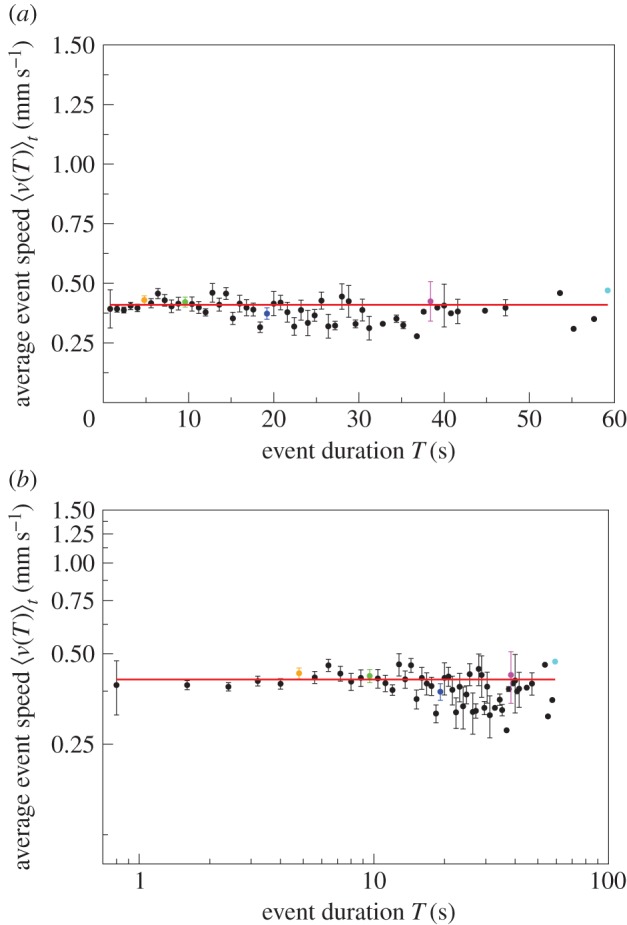
Data for 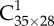 (solid circles) after random reallocation of speeds among the events, see null model, on (*a*) linear–linear scale and (*b*) log–log scale. Average event speed 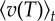 versus event duration *T*. Error bars indicate 1 s.e. of the mean. The longer the event duration, the bigger the error bars owing to fewer events. Data points without an error bar consist of just one event. The average event speed is constant, independent of the event duration. Fitting the randomized data yields *a* = 0.41 mm s^−1^, *β* = 0.

This procedure removes any correlations between the speeds and the events. Indeed, in the null model the average speed is constant and independent of the event duration (figures 7 and 8).

**Figure 9.**
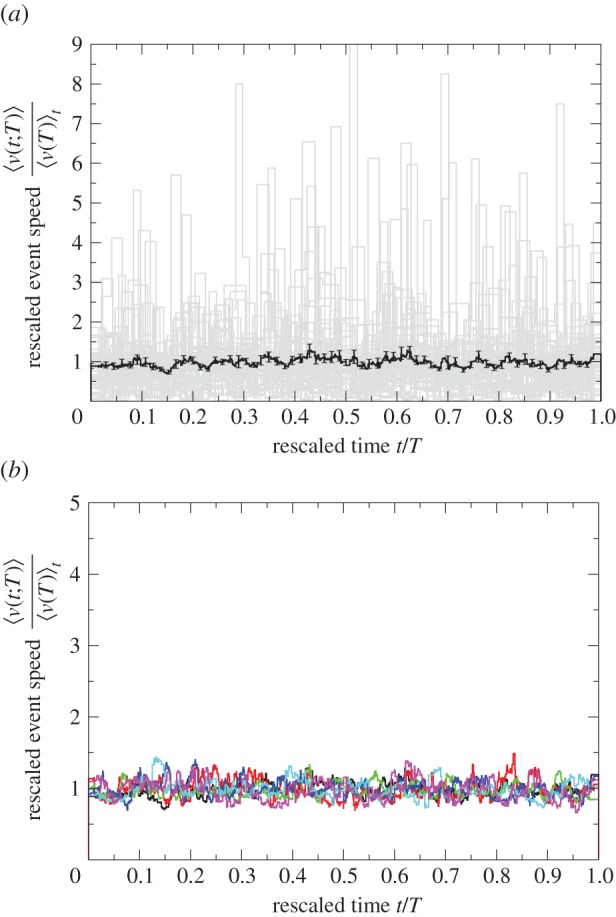
Data randomized according to the null model. (*a*) Data for colony 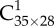; black line shows the mean rescaled event speed profile over all event durations; grey lines represent the rescaled event speed profiles for the different event durations. Error bars indicate one standard error of the mean. (*b*) All three colonies in each of the two nest sizes. The event speed has been rescaled with 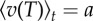 with *a* = 0.4088, 0.4925, 0.3265, 0.6130, 0.3475, 0.6010 mm s^−1^ for colonies and nest sizes 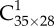, 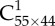, 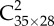, 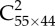, 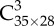, 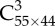, respectively, which represent the average speeds associated with that colony and nest size. Note that the rescaling here is based on a constant, 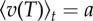, and is thus independent of event duration in contrast to the original data, where 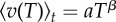. Furthermore, the acceleration and deceleration at the beginning and end, respectively, of an event are absent.

Furthermore, the initial acceleration and the final deceleration disappear and the universal function is constant ≈1 (figure 9).

## 7. Discussion and conclusion

**Figure 10.**
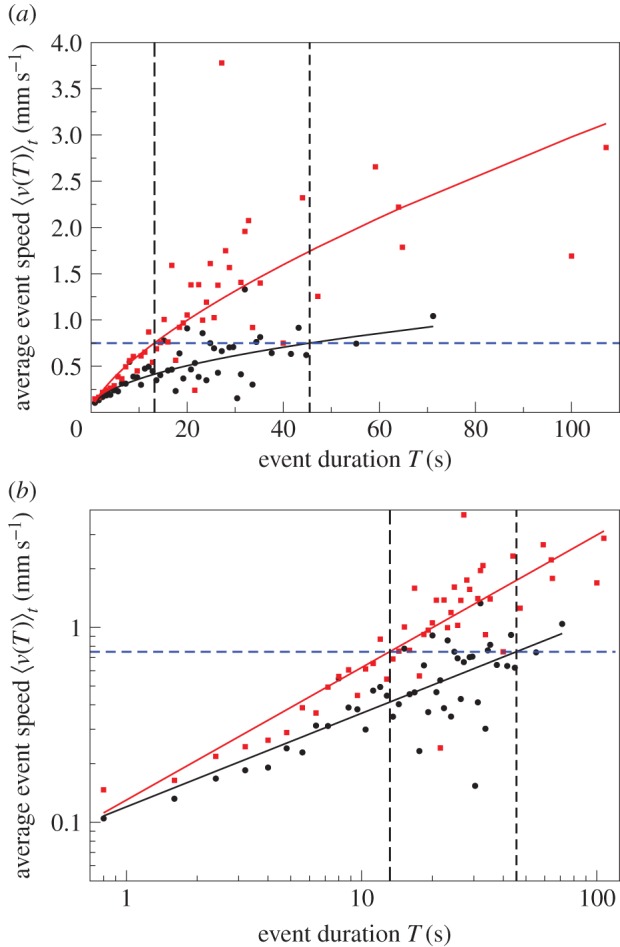
Illustration of the argument that the arrow of causation points in the direction from event duration to event speed. The data are for C^3^ on (*a*) linear–linear scale and (*b*) log–log scale; black circles—small nest size: 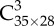; red squares—large nest size: 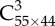; black and red lines represent the line of best fit from the linear regression in the electronic supplementary material, figure S1 (the values of the parameters *a* and *β* are as in table 2, columns six and seven for the small and large nest size, respectively). The value of average event speed highlighted in blue is the mean of 0.75 mm s^−1^ over all treatments and colonies. When the colony is in the large nest, the average event speed of 0.75 mm s^−1^ corresponds to event duration of 13.2 s. By contrast, the same average event speed of 0.75 mm s^−1^ corresponds to event duration of 45.5 s when the same colony is in the small nest. In other words, the same average event speed is associated with a longer event duration in the smaller nest. This contradicts the idea that speed determines event duration because ants moving at the same speed should move for longer in the large nest where there is more space. Therefore, we favour the alternative, namely that the event duration is already specified when the ant begins to move and it reaches a higher speed in the larger nest because there is more free space available.

What is the origin of the relationship between the average event speed 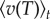 and the event duration *T*? Possibly, the more the free space in which an ant can move, the higher the speed, on average, the ant can reach and this in turn determines the duration *T* of the activity. Alternatively, an ant may have a pre-determined duration *T* of a movement event and may adjust its speed as a consequence of event duration. This second alternative appears less probable because it would require ants to have an internal mechanism, which determines the duration of their movement events. However, the relationships in figures 1 and 2 provide evidence for the second alternative. The speed and the event speed profiles clearly demonstrate that ants occasionally reach very low speeds during a movement event. This suggests that an ant does not necessarily stop after it has reduced its speed. Indeed, the arrow of causation with speed as the determinant of event duration is eliminated by the relationship between event duration and average event speed for the two nest sizes. According to this relationship, the event duration for a given average event speed is longer in the smaller nest (figure 10). This contradicts the idea that when there is more free space, the speed an ant can reach leads to a longer event duration. Therefore, we favour the arrow of causation which points from duration to average speed in the processes underlying activity events in ants.

We found that the exponent of the power-law relationship between average event speed and event duration is larger in the larger nests. This means that for any event duration the average event speed of an ant, on average, is higher when its colony resides in the larger nest and that this difference increases disproportionately with increasing event duration. Such a nonlinear effect suggests interactions. Indeed, everything else being equal, when a colony resides in the smaller nest, there is a higher probability of interactions with other colony members due to the higher density of workers and brood per unit area. This in turn is likely to reduce the average speed associated with the respective event duration. Hence our results also reveal that the universal relationship between activity event duration and average speed is flexible to meet the requirements of a growing colony. The two nests we used were of a medium size, that is on average 2000 mm^2^, with a range between 841 and 3025 mm^2^, the area preferred by both large and small *T. albipennis* colonies, which typically grow to a size of 400 workers [22]. Furthermore, none of the colonies in our study had their space restricted. Even workers in the largest colony (121 workers; table 1) in the smaller nest had more space than the 5 mm^2^ per adult worker provided by *T. albipennis* colonies when they build their own encircling nest wall out of sand grains [23].

We studied the bouts of activity of individual ants within their complete societies inside laboratory nests. Therefore, the relationship between activity event duration and average speed could be the result of individual behaviour, social interactions or a combination of the two. To establish the importance of social interactions for this relationship, we suggest that future studies use manipulative experiments or mutual information approaches to larger datasets [24,25].

Our results are based on the activity of ants but we are convinced that our main conclusion that the duration of an activity event is determined before it commences is likely to be applicable as a general principle of animal behaviour across taxa, including humans. As our results also demonstrate, such a principle is not fixed and works in a feedback loop with the environment. Furthermore, the colonies in our experiment were in everyday, static conditions. If these conditions are perturbed and the system is under stress, things could change. Such hypotheses should be tested in future experiments using the generic framework applied here. This will elucidate further the underlying causal relationships in the way biological social systems work and inform the engineering and control of artificial social systems.

## Supplementary Material

ELECTRONIC SUPPLEMENTARY INFORMATION

## Data accessibility

Data are available at doi:10.5523/bris.cmcs6znssfim12zo6zzmur1hq.
