# Supplementary material for: Universality in ant behaviour
Source: J R Soc Interface. 2015 Jan 6;12(102):20140985. doi: 10.1098/rsif.2014.0985 (PMC4277088; doi:10.1098/rsif.2014.0985)
Supplement: ELECTRONIC SUPPLEMENTARY INFORMATION [file rsif20140985supp1.pdf]

ELECTRONIC SUPPLEMENTARY MATERIAL

Universality in Ant Behaviour

Kim Christensen, Dario Papavassiliou, Alexandre de Figueiredo, Nigel R. Franks, Ana B. Sendova-Franks

Statistical analysis

We tested whether the linearized relationship between event duration and average event speed has a significantly different gradient (the exponent in the non-linearized relationship) for the two different nest sizes (Fig. S1). We applied a General Linear Mixed Model using the function lmer() in the R package 'lme4' [22]. Colony was treated as a random factor (Fig. S2). Goodness-of-fit measures for the final model are presented in Figs S3-S5. The model selection process and the final model with its interpretation are presented in Tables S1 and S2, respectively.

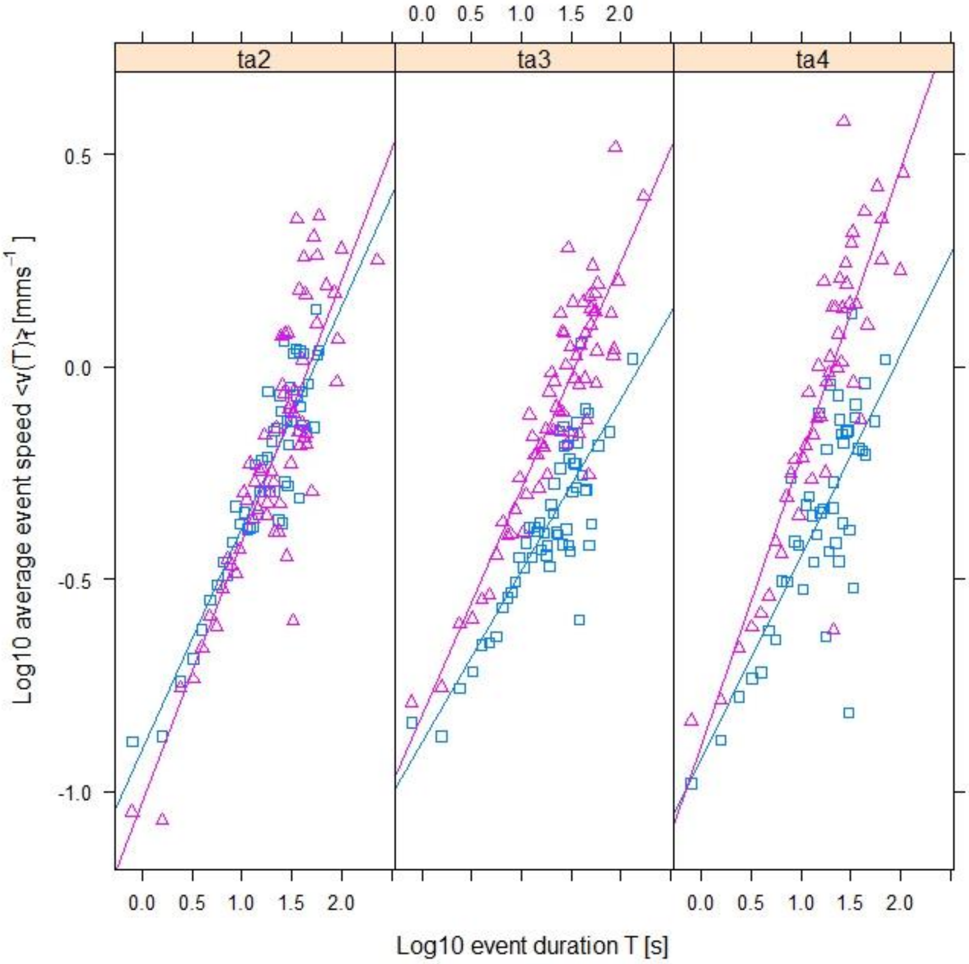

Figure S1: Relationship between log10 event duration [s] and log10 average event speed [mms^{-1}] for each colony for each of the two nest sizes. The labels 'ta2', 'ta3' and 'ta4' denote colonies 1, 2 and 3, respectively. Blue squares stand for nest size 35 x 28 mm^2, purple triangles denote nest size 55 x 44 mm^2. Blue and purple lines are simple linear regression lines for the respective nest size. Please note that colony 1 ('ta2') was the largest (Table I), which would have made less of a difference between the small and large nest sizes than in the other two colonies. This could explain the much smaller effect of nest size on the relationship between log10 event duration and log10 average event speed in this colony.

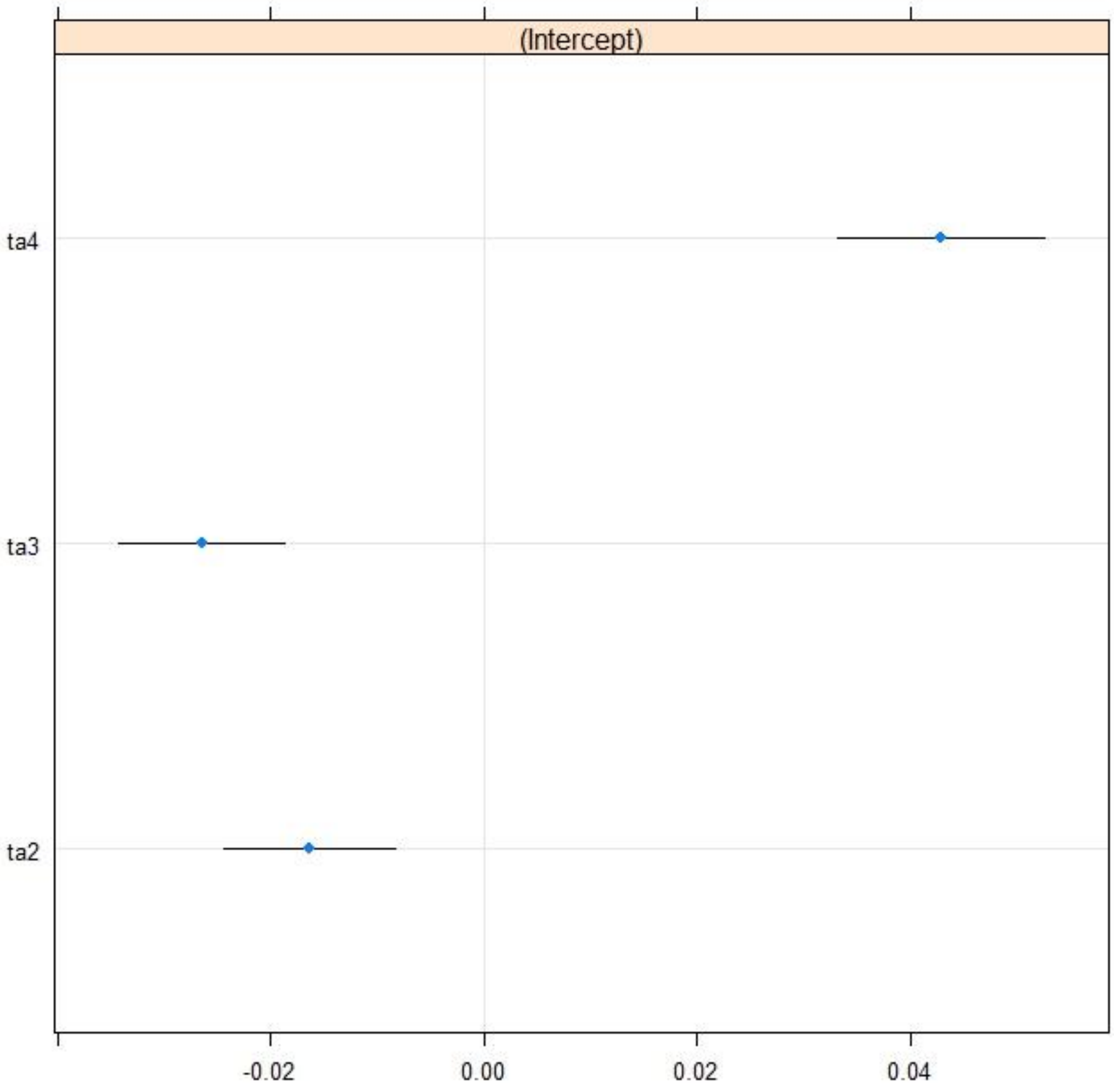

Figure S2: A ‘caterpillar’ plot for the effect of the random factor ‘colony’ in the null model, 0 (Table S1). The labels ‘ta2’, ‘ta3’ and ‘ta4’ denote colonies 1, 2 and 3, respectively. None of the 95% prediction intervals (PIs) for the three colonies straddles 0. This means that all three colonies deviate significantly from the overall intercept and hence the inclusion of the random factor ‘colony’ in the model is necessary. Therefore, applying a Linear Mixed Model rather than a Linear Model is justified.

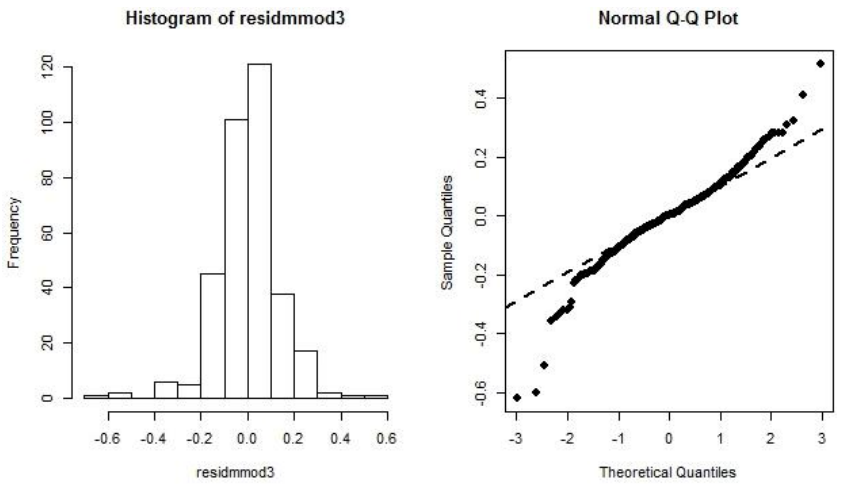

Figure S3: The overall residuals for the selected, final, model (model 3, Table S1) are approximately normal with a little deviation from normality in the tails. The Shapiro-Wilk's test shows a significant deviation from normality (W = 0.9539, p-value = 7.756e-09). This is to be expected to some extent for a sample size of 340 activity events and the model is robust to some deviation from normality.

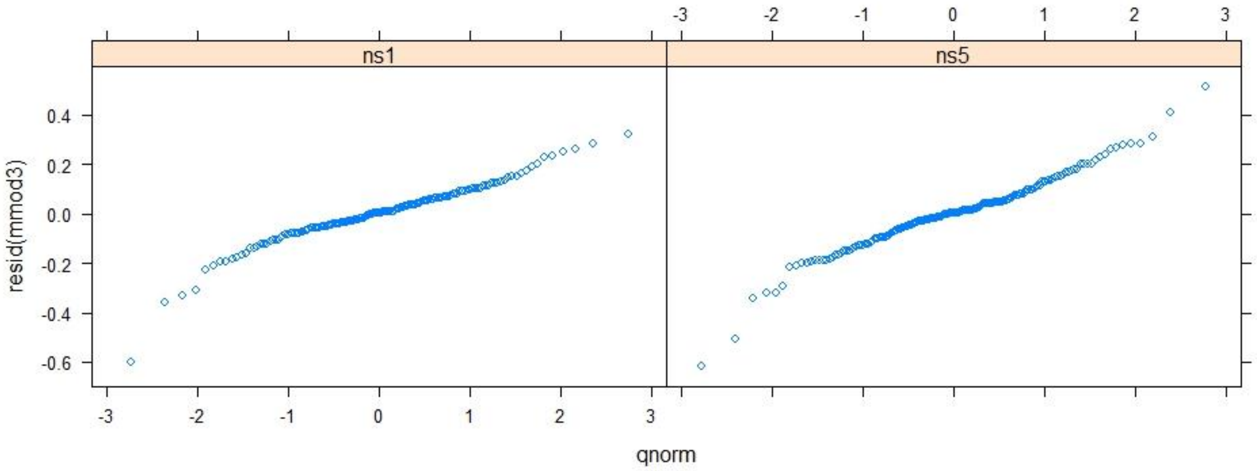

Figure S4: A Q-Q plot for the final model's residuals per nest size (ns1: 35 x 28 mm^2 and ns5: 55 x 44 mm^2) reveals that most of the deviations from normality are in the extreme tails particularly for the large nest size.

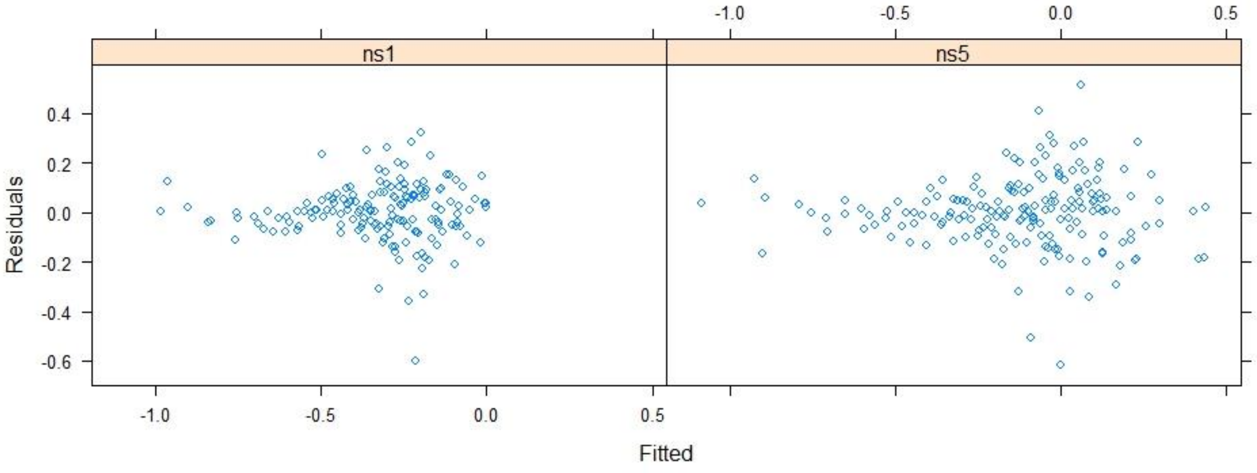

Figure S5: An examination of residuals-versus-fitted-values plots per nest size for the final model reveals that the residuals are approximately homogeneous with a little tale towards negative values particularly for the large nest size (ns1: 35 x 28 mm^2 and ns5: 55 x 44 mm^2).

Model	Random effects	Df	AIC	BIC	logLik	Chi-sq	Chi Df	Pr(>Chisq)
0	*(1|colony)*	*6*	*-320.52*	*-297.54*	*166.26*			
1	(nest size|colony)	8	-367.97	-337.34	191.99	51.453	2	<0.0001
2	(event duration|colony)	8	-320.08	-289.44	168.04	0.000	0	1.0000
**3**	**(nest size|colony) + (-1+event duration|colony)**	**9**	**-371.36**	**-336.90**	**194.68**	**53.282**	**1**	**<0.0001**
4	(nest size|colony) + (-1+event duration|colony) + (-1+nest size:event duration|colony)	12	-365.82	-319.88	194.91	0.465	3	0.9266

Table S1: Model selection: the five compared models differ only in their random effects component, which involves the random factor ‘colony’. The response variable in each model is the log10 average event speed [mms^{-1}] (Fig. S1). The fixed component consists of log10 event duration [s], the effect of nest size (35 x 28mm^2 or 55 x 44 mm^2) and the interaction between log10 event duration and nest size (Fig. S1). Any effect of log10 event duration would be represented by a non-zero slope in its relationship with log10 average event speed. Any effect of nest size would be represented by differences between the intercepts of the two lines in each plot of Figure S1. Any effect of the interaction between nest size and log10 event duration would be represented by differences between the slopes of the two lines in each plot of Figure S1. All five models were fitted with the lmer() function in the R package ‘lme4’[22] and compared with the anova() function, which uses a Likelihood Ratio Test (LRT) to evaluate models fitted with lmer(). A significant Chi-sq test statistic from the LRT indicates that the extra term in a subsequent model contributes significantly. A smaller value for AIC or BIC indicates a better fit of the model to the data. Model 0 is the null model with the random factor ‘colony’ allowed to vary around the overall intercept (average value). In Model 1, the random factor ‘colony’ is allowed to vary simultaneously around the overall intercept and around the individual intercepts for each nest size. In Model 2, the random factor ‘colony’ is allowed to vary simultaneously around the overall intercept and around the overall slope of the relationship between log10 average event speed and log10 event duration. In Model 3, the random factor ‘colony’ is allowed to vary simultaneously around the overall intercept, the individual intercepts for each nest size and the overall slope of the relationship between log10 average event speed and log10 event duration. Finally, Model 4, is the same as Model 3 with the additional allowance for the random factor ‘colony’ to vary around the individual slopes for each nest size in the relationship between log10 average event speed and log10 event duration. Model 3 is the selected, final, model. The ΔAIC between model 3 and 1 is 3 and on that basis there should be nothing to distinguish between these two models. However, when model 1 and 3 are compared directly, the chi-sq value for the LRT is 5.3877 and for 1 d.f. that corresponds to p = 0.02028, which is significant at the 5% significance level. This offers some evidence that model 3 is better than model 1. Hence model 3 was chosen as the best model (see Table S2 for interpretation).

```
Linear mixed model fit by REML ['lmerMod']
Formula: log10MeanSpeed ~ log10EventDuration * NestSize + (NestSize | Colony) +
(-1 + log10EventDuration | Colony)
Data: slopes

REML criterion at convergence: -369.2247

Random effects:
 Groups   Name               Variance Std.Dev. Corr
 Colony   (Intercept)        0.001945 0.04410
          NestSizens5        0.021995 0.14831*  -1.00
 Colony.1 log10EventDuration 0.001028 0.03205
 Residual                    0.017882 0.13372
Number of obs: 340, groups: Colony, 3

Fixed effects:
                                Estimate Std. Error t value
(Intercept)                    -0.903521   0.042413 -21.303
log10EventDuration              0.467489   0.031956  14.629
NestSizens5                    -0.003336   0.097315  -0.034#
log10EventDuration:NestSizens5  0.135404   0.034619   3.911◊
```

Table S2: The chosen model 3 run with treatment contrasts using the smaller nest size, 35 x 28 mm^2, as the base. The values highlighted in bold are the intercept and slope for the relationship between log10 average event speed [mms^{-1}] and log10 event duration [s] for the smaller nest size, 35 x 28 mm^2, averaged over all three colonies. The underlined values could be used to calculate the intercept and slope for the same relationship for the bigger nest size, 55 x 44 mm^2: intercept = **-0.903521**-0.003336 = *-0.906857*; slope = **0.467489+**0.135404 = *0.602893*. The estimated values for a (the constant) and β (the exponent) for the smaller nest size, 35 x 28 mm^2, are 10^(-0.903521) = 0.1248760 and 0.467489, respectively. The estimated values for a and β for the bigger nest size, 55 x 44 mm^2, are 10^(-0.906857) = 0.1239205 and 0.602893, respectively. Most of the random variation for colonies is around the intercept for the large nest size, 55 x 44 mm^2, relative to the small nest size, 35 x 28 mm^2 *: Std. Dev. = 0.14831 (see also Fig. S1). There is no significant difference between the intercepts (and hence between the a values) for the two nest sizes over all colonies (#:t=-0.034). There is a significant difference between the slopes (and hence between the β values) for the two nest sizes over all colonies (◊ : t=3.911; given the sample size of 340, z-values of 1.96, 2.576 and 3.291 could be used as critical values for 5%, 1% and 0.1% significance level respectively, and 3.911 is greater than each of them). The greater nest size, 55 x 44 mm^2, has the greater slope and hence the greater exponent for the sub-linear increase in average event speed with event duration, $\langle v(T) \rangle_t = aT^{\beta}$.

Robustness of the results

To test the robustness of the results we changed the time unit on which we based our definition of an *activity event*. We defined an *activity event* as an interval with non-zero instantaneous speed delineated by time units with zero speed at the beginning and end. The time unit in the main analysis is 0.8 s. In addition, we analysed the results with unit intervals of 0.2 s, 0.4 s, 1.6 s and 3.2 s. We carried the same statistical analyses on the data based on each of the above five time units.

We found that the results are robust to a five-fold doubling of the original time unit of 0.1 s available from the tracking software. The exponent β tended to increase with increasing time unit but so did its 95% confidence interval (Table S3). By contrast, the coefficient *a* tended to decrease with increasing time unit and so did its 95% confidence interval (Table S3).

(a)

Colony	C^1_{35x28}	C^1_{55x44}	C^2_{35x28}	C^2_{55x44}	C^3_{35x28}	C^3_{55x44}
**Exponent β**	0.44	0.51	0.34	0.40	0.34	0.54
**95% CI for β**	±0.07	±0.08	±0.07	±0.06	±0.06	±0.08
**Coefficient a**	0.70	0.66	0.69	1.01	0.62	0.82
**95% CI for a**	±0.09	±0.11	±0.08	±0.13	±0.06	±0.11

(b)

Colony	C^1_{35x28}	C^1_{55x44}	C^2_{35x28}	C^2_{55x44}	C^3_{35x28}	C^3_{55x44}
**Exponent β**	0.49	0.57	0.38	0.51	0.44	0.64
**95% CI for β**	±0.07	±0.08	±0.07	±0.07	±0.08	±0.09
**Coefficient a**	0.31	0.27	0.31	0.39	0.29	0.31
**95% CI for a**	±0.05	±0.06	±0.05	±0.07	±0.05	±0.07

(c)

Colony	C^1_{35x28}	C^1_{55x44}	C^2_{35x28}	C^2_{55x44}	C^3_{35x28}	C^3_{55x44}
**Exponent β**	0.52	0.61	0.40	0.53	0.48	0.68
**95% CI for β**	±0.06	±0.09	±0.07	±0.06	±0.12	±0.11
**Coefficient a**	0.13	0.10	0.13	0.15	0.12	0.13
**95% CI for a**	±0.03	±0.03	±0.03	±0.04	±0.04	±0.04

(d)

Colony	C^1_{35x28}	C^1_{55x44}	C^2_{35x28}	C^2_{55x44}	C^3_{35x28}	C^3_{55x44}
**Exponent β**	0.50	0.63	0.38	0.51	0.53	0.66
**95% CI for β**	±0.11	±0.09	±0.08	±0.11	±0.11	±0.16
**Coefficient a**	0.05	0.04	0.06	0.07	0.05	0.05
**95% CI for a**	±0.03	±0.01	±0.02	±0.03	±0.02	±0.04

(e)

Colony	C^1_{35x28}	C^1_{55x44}	C^2_{35x28}	C^2_{55x44}	C^3_{35x28}	C^3_{55x44}
**Exponent β**	0.58	0.62	0.41	0.57	0.52	0.65
**95% CI for β**	±0.12	±0.16	±0.11	±0.12	±0.14	±0.17
**Coefficient a**	0.02	0.01	0.03	0.03	0.02	0.02
**95% CI for a**	±0.01	±0.02	±0.01	±0.02	±0.01	±0.02

Table S3: Parameters for the power-law relationship $\langle v(T)\rangle_t = aT^{\beta}$ for a definition of *activity event* based on different time units: (a) 0.2 s, n = 332; (b) 0.4 s, n = 325; (c) 0.8 s, n = 340 (same as Table II in main text and shown here for completeness); (d) 1.6 s, n = 329 and (e) 3.2 s, n = 309.

The statistical modelling of the log10 average event speed [mms^{-1}] as a response to log10 event duration [s], nest size (35 x 28mm^2 or 55 x 44 mm^2), the interaction between the two and the effect of the random factor 'colony' (Fig. S1) identified model 1 and 3 (Table S1) as the best models for each of the five time units. Model 3 was better than model 1 for time units 0.8 s and 1.6 s but in all cases the interaction between log10 event duration [s] and nest size was significant such that the slope, and hence the exponent β, was significantly greater for the larger nest size (Table S4).

Time unit (s)	Model 1	Model 3	n
**0.2**	3.445	3.490	332
**0.4**	4.213	4.236	325
**0.8**	3.878	3.911	340
**1.6**	2.885	2.884	329
**3.2**	2.146	2.116	309

Table S4: Values of the t-statistic for testing the hypothesis that the slope, and hence the exponent β, for the relationship between log10 average event speed [mms^{-1}] and log10 event duration [s] (Fig. S1) is the same for both nest sizes for the two best models, 1 and 3 (Table S1). Given the sample sizes are between 309 and 340, the z-values of 1.96, 2.576 and 3.291 could be used as critical values for the 5%, 1% and 0.1% significance level, respectively. This means that the null hypothesis that the exponent β is the same for both nest sizes can be rejected at the 0.1% significant level for the 0.2 s, 0.4 s and 0.8 s time units, at the 1% significance level for 1.6 s time unit and at the 5% significance level for the 3.2 s time unit.

References:

[22] Bates, D., Maechler, M. & Bolker, B. (2014) lme4: Linear mixed-effects models using Eigen and S4. R package version 1.0-6. http://CRAN.R-project.org/package=lme4.
